# The study of Nickel Resistant Bacteria (NiRB) isolated from wastewaters polluted with different industrial sources

**DOI:** 10.1186/2052-336X-12-44

**Published:** 2014-01-29

**Authors:** Hoda Alboghobeish, Arezoo Tahmourespour, Monir Doudi

**Affiliations:** 1 Microbiology Department, Falavarjan Branch, Islamic Azad University, Falavarjan, Isfahan, Iran; 2 Basic Medical Sciences Department, Khorasgan (Isfahan) Branch, Islamic Azad University, Isfahan, Iran

**Keywords:** Nickel resistant bacteria (NiRB), Maximum tolerance concentration, Multiple metal resistance determination, 16SrDNA, Wastewater

## Abstract

**Background:**

Pollution due to the heavy metals is a problem that may have negative consequences on the hydrosphere. One of the best procedures in removing the toxic metals from the environment is using metal resistant bacteria.

**Results:**

In the present study eight nickel resistant bacteria were isolated from industrial wastewaters. Three of them were selected as the most resistant based on their Maximum tolerable concentration (8, 16 and 24 mM Ni^2+^). Their identification was done according to morphological, biochemical characteristics and 16SrDNA gene sequencing and they were identified as *Cupriavidus sp* ATHA3, *Klebsiella oxytoca* ATHA6 and *Methylobacterium sp* ATHA7. The accession numbers assigned to ATHA3, ATHA6 and ATHA7 strains are JX120152, JX196648 and JX457333 respectively. The Growth rate of the most resistant isolate, *Klebsiella oxytoca strain ATHA6,* in the presence of Ni^2+^ and the reduction in Ni^2+^ concentration was revealed that *K oxytoca ATHA6* could decrease 83 mg/mL of nickel from the medium after 3 days.

**Conclusion:**

It can be concluded that the identified Ni resistant bacteria could be valuable for the bioremediation of Ni polluted waste water and sewage.

## Background

The pollution of the environment with toxic heavy metals is increasing throughout the world along technological development. Copper, Chromium, Cadmium and Nickel are known to be the most commonly heavy metals used and the more wide spread contaminants of the environment [1,2]. Wastewater contains significant concentration of heavy metals that are not degraded by the conventional process of wastewater treatment. The main source of heavy metals is the industrial activities such as metal processing, mining and electroplating, tanning, carpet washing and dying. Presence of high concentration of toxic heavy metals in waste water can cause severe problems to human health [3]. Bioremediation can be used to effectively reduce contaminant toxicity, mobility or volume to levels that are innocuous to human health and ecosystem [4]. Microorganisms possess mechanisms that regulate metal ion accumulation to avoid heavy metal toxicity and there are many reports about microbial resistance to heavy metals [5-7]. To survive under metal- stressed conditions, bacteria have evolved several types of mechanisms to tolerate the uptake of heavy metal ions. These mechanisms include the efflux of metal ions outside the cell, accumulation of the metal ions inside the cell and reduction of the heavy metal ions to a less toxic state [8].

Nickel is the 24th most abundant element in the earth crust [9]. Microorganisms have evolved in the presence of this metal, which is necessary in trace amounts for a variety of metabolic processes but in high concentration causing oxidative stress in the cell. The best known mechanisms of nickel resistance are mediated by efflux pumps such as cnr CBA (cobalt- nickel resistance) from *Cupriavidus metallidurans* CH34 (formerly *Ralstonia metallidurans* CH34), NccCBA (Nickel-cobalt-cadmium) and NreB (nickel resistance) from *Achromobacter xylosoxidans*31A, CznABC (Cadmium-zinc-nickel) from *Helicobacter pylori*[10]. The aims of this study are isolation and characterization of nickel resistant bacteria (NiRB) from industrial wastewater, MTC (maximum tolerable concentration) and multiple metal tolerance determination of them.

## Materials and methods

### Sampling

Wastewater samples were collected in screw capped sterilized bottle from Factory’s steel- copper- smithy- sewage treatment plant of Isfahan (Iran). Some physicochemical parameters of wastewater viz., temperature (°C), pH, BOD, COD were measured [11].

### Isolation and screening of Ni-resistant bacteria

Wastewater sample was spread on PHGII agar Plates containing 0.5 mM of *Ni*^2+^. PHGII agar plates were prepared by dissolving 2 g NaCl, 4 gr peptone and 1gr yeast extract in 1000 mL distilled water, pH was adjusted at 7.0 and then 15 gr agar was added in the 1000 mL Flasks. The medium was autoclaved at 121°C and 15 Lb pressure for 15 minutes, then supplemented with *Ni*^2+^ (0.5 mM/L). The growth of bacterial colonies was observed after 5 days of incubation at 30°C [12].

### Identification of the bacterial isolate

Selected isolates were grown on LB (Luria- bertani) media. The shape and color of the colonies were examined under the Microscope after gram staining. Isolates were biochemically analyzed for the activities of oxidase, Catalase, VP-MR test, motility, indole production and citrate utilization according to Bergey’s manual of systematic Bacteriology [13].

### Determination of maximum tolerable concentration and multi metal resistance

The Maximum Tolerable Concentration of heavy metal was selected as the highest concentration of heavy metal that allows growth after 2 days [14]. The increasing concentration of Ni (1, 2, 4, 8, 16, 24, 32 mM) on PHGII agar plates were used for testing the MTCs of NiRB. Then multi metal resistances of selected bacteria were also investigated [15].

### 16 SrDNA gene amplification and sequencing

Molecular identification of the most NiRB was performed by 16S rDNA PCR Sequencing [16]. Genomic DNA was extracted and the 16S rDNA gene was amplified by using the universal bacterial Primers of F27 (5′-CAGGGTACCAGAGTTTGA-3′) andR1492 (5′-CTCTCTGCAGTACGGCTAC-3′).

PCR was performed as a 25 μL reaction Mixture containing 2 μL of DNA extract as a template. Each primer at a concentration of 8 PM, 2 mM mgCl_2_ and dNTP_S_ at a concentration of 0.2 mM, as well as 1.25 U of Taq polymerase and buffer were used. After the initial denaturation for 5 min at 95°C, there was 32 Cycle consisting of denaturation at 94°C for 1 min, annealing at 57°C for 1 min, extension at 72°C for 1 min and final extension at 72°C for 5 min. PCR was carried out in a gene AMP PCR system (Eppendorf). PCR products were analyzed by 1% (W/V) agarose gel electrophoresis in 0.5x TBE buffer with ethidium bromide (05 μ_g_/ml). PCR products were then sequenced and compared with the National Center for Biotechnology Information (NCBI) database using the BLAST search available through the center’s website (http://www.ncbi.nlm.nih.gov/BLAST). The 16S rDNA sequences were then submitted to the Gene Bank using the BankIt service.

### Nickel removal by isolated bacterium

Growth of selected isolate was carried out in PHGII broth supplemented with (12 mmol Ni^2+^/mL) and without Nickel (control).

The cultures were incubated at 30°C for 6 days and from each medium (control and treated) the amount of 5 ml was taken out under sterilized conditions after 24, 48, 72, 96, 144, 168 and 216 hours, respectively. The cultures were centrifuged at 3000 rpm for 5 minutes and supernatants were used for the estimation of Ni by atomic absorption Spectrophotometer (a Perkin-Elmer 800 atomic absorption spectrophotometer).

## Results

The physicochemical characteristics of waste waters are shown in Table 1. From the three studied industrial wastewater, eight Ni resistant bacteria were isolated. The biochemical characteristics of isolated bacteria were shown in Table 2.

**Table 1:** Physicochemical characteristics of waste waters

**Waste waters**	**Temperature (°C)**	**pH**	**COD mg/L**	**BOD**_**5 **_**mg/L**
Factory’s steel	28	8.4	70	18
Sewage treatment plant	29	7.4	182	34
Copper smithy	28	8.3	123	25

**Table 2:** Morphological and Biochemical characteristics of bacteria

**Bacteria**	**S1**	**S2**	**S3**	**S4**	**S5**	**S6**	**S7**	**S8**
Morphological								
Cell morphology	Cocci	Rod	Rod	Cocci	Rod	Cocci	Rod	Rod
Gram reaction	−	−	−	−	−	+	−	−
Motility	+	−	−	+	+	−	+	−
Biochemical								
Catalase	+	+	+	+	+	+	+	+
Oxidase	+	−	+	+	+	−	+	−
Indole	−	−	+	−	−	ND	−	+
VP	ND	+	−	ND	+	ND	+	+
MR	ND	−	+	ND	−	ND	−	−
Citrat	+	−	+	+	+	ND	−	+
Nitrat	+	−	+	+	+	+	+	+
Utilization of								
Manitol	−	−	+	−	−	+	−	+
Glucose	−−	−	+	−	−	+	−	+
Fructose	−	−	+	−	−	+	−	+
Lactose	−	−	−	−	−	ND	−	+
Results	Moraxella (Bovis)	Acinetobacter (Lwoffi)	Providencia (stuartii)	Branhamella (Catarhalis)	Cupriavidus sp ATHA3	S. aureus	Methylobacterium sp ATHA7	Klebsiella oxytoca ATHA6

Symbols: (−): Negative; (+): Positive; (ND): not determined.

**Figure 1:**
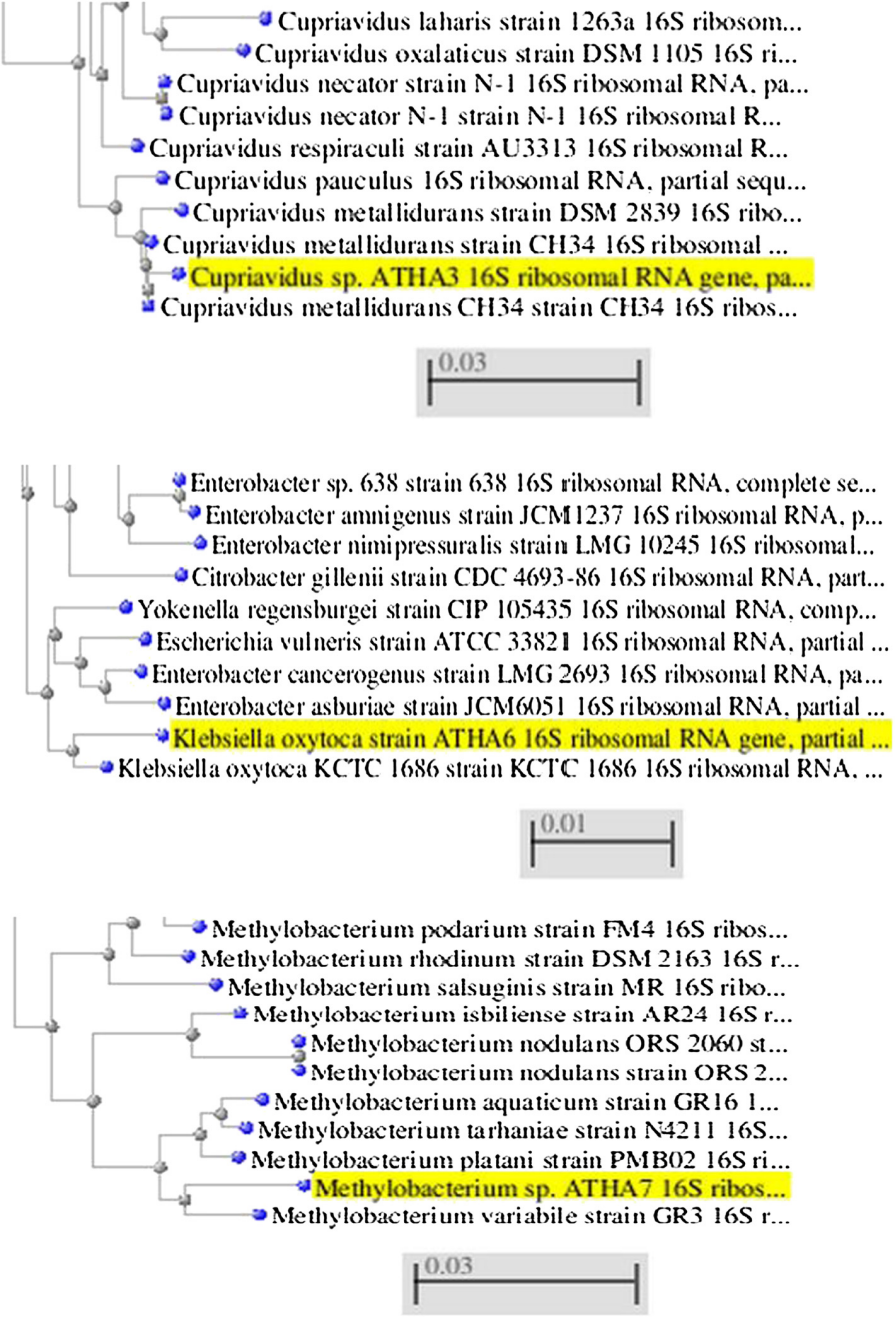
The phylogenetic evidences of identified strains of ATHA3, ATHA6 and ATHA7.

Maximum Tolerable Concentration of Ni- resistant bacteria were shown in Table 3 and three isolates were selected as the most resistant (S5, S7 and S8). Then the Multi metal resistant of the 3 selected isolates was determined and are shown in Table 4. Also, the gene sequence analysis were done to further identify the bacteria and to support the results from the biochemical analysis of the most resistant isolates. The phylogenetic distance trees of results are shown in Figure 1. The percentage of maximum similarity and GenBank accession number are shown in Table 5.

**Table 3:** Maximum tolerable concentration of Ni

**Code**	**Bacteria**	**MTC Ni**^**2+ **^**(mM)**
S1	*Moraxella (Bovis)*	1
S2	*Acinetobacter (lwoffi)*	2
S3	*Providencia stuaratii*	2
S4	*Branhamella (catarhalis)*	2
S5	*Cupriavidus sp ATHA3*	8*
S6	*Staphylococcus (aureus)*	2
S7	*Methylobacterium sp ATHA7*	16*
S8	*Klebsiella oxytoca ATHA6*	24*

*: The stared strains were selected as the most resistant isolates.

**Table 4:** Multimetal resistance of the most resistant bacteria

**Metals (mM) bacteria**	**Pb**	**Cd**	**Cu**
*Klebsiella oxytoca ATHA6*	2	-	1
*Methylobacterium sp ATHA7*	2	8	-
*Cupriavidus sp ATHA3*	2	8	2

**Table 5:** The percentage of maximum similarity and GenBank accession numbers of Ni resistant bacteria

**Isolate**	**Organism**	**Identity (%)**	**Accession no.**
S5	*Cupriavidus sp ATHA3*	98% similarity to *Cupriavidus metalidurans* strain CH34	JX120152
S7	*Methylobacterium sp ATHA7*	96% similarity to *Methylobacterium Variabile* strain GR3	JX457333
S8	*Klebsiella oxytoca ATHA6*	99% similarity to *Klebsiella oxytoca*	JX196648

**Figure 2:**
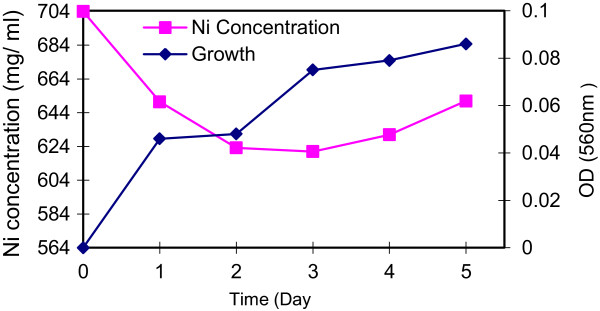
**Growth rate of *Klebsiella oxytoca ATHA6 *** **and reduction in Ni concentration.**

The Growth rate of the most resistant isolate, *Klebsiella oxytoca strain ATHA6,* in the presence of Ni^2+^ (704 mg/ml) and the reduction in Ni^2+^ concentration are shown in Figure 2. It is revealed that *K oxytoca ATHA6* could decrease 83 mg/mL of nickel from the medium after 3 day.

## Discussion

Heavy metal-resistant microorganisms are thought to naturally occur; primarily in metal-contaminated soils. Studies suggest that metals as well as other soil physicochemical parameters can contribute to bacterial resistance to metals [17]. Soil pH can influence the solubility of metals, [18]. The pH values of the soil samples revealed no significant differences at all localities and ranged from 7.4 to 8.4. Both the BOD and COD tests are a measure of the relative oxygen-depletion effect of a waste contaminant [19]. Both have been widely adopted as a measure of pollution effect. So according to results the studied waste water were belonged to weakly contaminant wastewaters.

Surviving microorganisms in a stressed environment especially in presence of heavy metals is dependent to development of tolerance mechanisms [20]. They also play important roles in the cycling of toxic metals in the biosphere. While toxic mechanism of all heavy metals is similar, multiple tolerances are common phenomena among heavy metal resistant bacteria. So, in this research efforts were made to isolate nickel resistant bacteria (NiRB) from industrial effluents.

Eight NiRB were isolated from studied industrial effluents. Most of them were Gram- negative cocci and bacilli; they were identified as *Moraxella (Bovis), Acinetobacter (Lwoffi), Providencia (stuartii), Branhamella (Catarhalis), Cupriavidus sp ATHA3, S. aureus, Methylobacterium sp. ATHA7, Klebsiella oxytoca ATHA6.*

Virender et al. [2] also isolated gram- negative NiRB isolates [2]. All the isolates of this research exhibited resistance with maximum tolerable concentration (MTC) for nickel ranging from 1 to 24 mM. Among them, three isolated strains of ATHA3, ATHA7 and ATHA 6 were found to have significant higher MTC than the other isolates (8, 16 and 24 mM respectively) and selected as the most Ni resistant isolates for further study. Sevgi et al. [21] isolated NiRB and their resistance was found to be in the range of 2.0-4.0 mM of NiCl_2_ while it has also been reported that nickel-resistant bacteria [21]. *Alcaligenes xylosoxidans*, isolated from a galvanization tank, tolerated relatively high concentrations of nickel (40 mM) [22].

Spatel et al. [6] also isolated Ni resistant *Pseudomonas fragi* from industrial effluent containing different concentrations of nickel sulfate. Well growth of isolate was observed in the medium containing up to 2.5 mmol/L of nickel [6]. Rajbanshi studied on heavy metal resistant bacteria in sewage treatment plant and could isolate nickel resistant Staphylococcus spp. and Bacillus spp., which showed MIC ranging from 150 μg/mL to 500 μg/ml [23]. Arundhati and Paul [24] also isolated the nickel-resistant bacterium Cupriavidus sp. which exhibited a typical inducible Ni resistance in Ni supplemented (1.0-10.0 mM) Tris-minimal medium. This strain could accumulate a maximum of 29.3 μM Ni/g protein after 48 h of growth in 5 mM Ni. They were stated that nickel resistance in Cupriavidus may be due to extracytoplasmic binding and accumulation coupled with expression of specific periplasmic proteins [24].

Multi Metal Tolerance Test of the three NiRB isolates indicated highest tolerance to Cd (8 mM) by ATHA3 and ATHA7 strains and lowest to Cu (1 mM) by *ATHA6.* Pb resistance in all cases was 2 mM. NiRB isolates of Sevgi et al. [21] study, showed multi metal resistance as follows: cobalt (20 mM), zinc (10 mM), cadmium (1 mM), and copper (1 mM). Malik also isolated a total of 70 bacterial isolates from industrial and agricultural soils and tested for their resistance against several heavy metals. They reported that the industrial isolates were more resistant to different metals than agricultural isolates. They showed that 88.8% of isolates from industrial soil were resistant to Ni, 80% to Cr, 82.8% Zn, and 71.4% to Cd. Multiple tolerances occur only to toxic compounds that have similar mechanisms underlying their toxicity [25]. Since all heavy metals are similar in their toxic mechanisms multiple tolerances are common phenomena among heavy metal resistant bacteria [26].

The effect of Ni concentration on the bio removal capacity and growth rate of ATHA6 is shown in the Figure 1. Another finding of metal presence in the culture medium was reduction in growth rate as compared with control. This has been explained that the exposed microorganism to metals stress deviate its energy from growth to maintenance of other functions as a greater demand of energy to resist metal toxicity [27]. Bio removal efficiency increased with time, and maximum efficiency was observed at 72 h of growth (11.78% reduction in Ni^2+^ concentration). Bio removal was negligible after this time. Specific surface properties and the physiological state of the microorganisms might have a role in metal uptake. High biomass production is also important for better bio removal.

For the phylogenetic analysis of the most resistant NiRB isolates, 16S rDNA genes were amplified as described before. About 1500 bp PCR products were used for DNA sequencing with the same forward and reverse primers as the PCR primers. The 16S rDNA gene sequences of ATHA3, ATHA6 and ATHA7 strains were submitted to GenBank. The accession numbers assigned to ATHA3, ATHA6 and ATHA7 strains are JX120152, JX196648 and JX457333 respectively.

Comparison of 16S rDNA gene sequences revealed that ATHA3 showed about 98% similarity to *Cupriavidus metalidurans* strain CH34. *Cupriavidus metallidurans CH34* is a β-proteobacteria adapted to metal contaminated environments. It has AtmA, a chromosomal ABC transporter, causing resistance to nickel [28].

As a result of a BLAST search, 99% similarity was observed between the 16S rDNA gene sequence of ATHA6 and *Klebsiella oxytoca.* Stoppel and Schlegel [29] found chromosomally coded Nickel resistance genes of *K. oxytoca* that were not found to occur in combination with any of the other known nickel resistance determinants [29]. So, the nickel resistance genes of *K. oxytoca* may encode a second type of nickel resistance which appears independently from other nickel resistance determinants.

96% similarity of pink pigmented ATHA7 and *Methylobacterium Variabile* strain GR3 was observed. A 96% similarity in 16S rDNA gene sequences is a low value for identification at species level. Therefore, ATHA7 may be a member of a novel bacterial species. Various pink-pigmented *Methylobacteria* were also obtained from the rhizosphere and endosphere of hyper accumulating plant Thlaspi goesingense grown in Redschlag, Austria. These strains were found to exhibit different multiple heavy metal resistance characteristics to Ni, Cd, Co, Zn and Cr [30]. Aboudrar et al. [31] also confirmed that, the NiRB strains could reduce the soil bioavailability of Ni and also induce promoting effects on the growth of plant. So, they stated that NiRB strains could serve as an effective metal-immobilizing and growth-promoting bioinoculant for plants in Ni-stressed soils [31].

## Conclusion

It can be concluded that there are several different heavy metal resistant bacteria in the contaminated environment that should be isolated and identified for further studies. The most NiRB isolated of this study are *Cupriavidus sp* ATHA3, *Klebsiella oxytoca* ATHA6 and *Methylobacterium* sp ATHA7. These identified Ni resistant bacteria could be valuable for the bioremediation of Ni polluted waste water and sewage.

## Competing interests

The authors declare that they have no competing interests.

## Authors’ contributions

The overall implementation of this study including experiments, data analysis, and manuscript preparation were the results of joint efforts by individuals who are listed as co-authors of this paper. All authors read and approved the final manuscript.
